# Imaging findings in inflammatory disease of the genital organs

**DOI:** 10.1007/s11604-023-01518-8

**Published:** 2024-01-02

**Authors:** Saki Shibuki, Tsukasa Saida, Sodai Hoshiai, Toshitaka Ishiguro, Masafumi Sakai, Taishi Amano, Tetsuya Abe, Miki Yoshida, Kensaku Mori, Takahito Nakajima

**Affiliations:** 1 Department of Radiology, University of Tsukuba Hospital, 2-1-1 Amakubo, Tsukuba, Ibaraki 305-8576 Japan; 2 Department of Radiology, Institute of Medicine, University of Tsukuba, 1-1-1 Tennodai, Tsukuba, Ibaraki 305-8575 Japan

**Keywords:** Pelvic inflammatory disease, Actinomycosis, Tuberculosis, Genital organ

## Abstract

This review focuses on inflammatory diseases of female and male genital organs and discusses their epidemiology, pathogenesis, clinical presentation, and imaging findings. The female section covers pelvic inflammatory disease (PID) primarily caused by sexually transmitted infections (STIs) that affect the uterus, fallopian tubes, and ovaries. Unusual causes such as actinomycosis and tuberculosis have also been explored. The male section delves into infections affecting the vas deferens, epididymis, testes, prostate, and seminal vesicles. Uncommon causes such as tuberculosis, and Zinner syndrome have also been discussed. In addition, this review highlights other conditions that mimic male genital tract infections such as vasculitis, IgG4-related diseases, and sarcoidosis. Accurate diagnosis and appropriate management of these inflammatory diseases are essential for preventing serious complications and infertility. Imaging modalities such as ultrasound, magnetic resonance imaging, and computed tomography play a crucial role in diagnosis. Understanding the diverse etiologies and imaging findings is vital for the effective management of inflammatory diseases of the genital organs.

## Introduction

Pelvic inflammatory disease (PID) is a term used to describe infections that primarily affect female reproductive organs. It is commonly caused by sexually transmitted infections (STIs) and is a significant condition in women of reproductive age. In men, infections affecting the genital organs can spread retrogradely from the ejaculatory ducts or urethra to the epididymis, and from the urethra to the prostate, and seminal vesicles. These infections may arise because of STIs or urinary tract infections. Inflammatory diseases, whether in women or men, can lead to severe complications such as abscesses, peritonitis, and infertility. Therefore, early diagnosis and appropriate treatment are crucial. Understanding the diverse etiologies and manifestations of genital infections is important for accurate diagnosis. This article aims to provide a comprehensive overview of inflammatory diseases of the genital organs, including their epidemiology, pathogenesis, clinical presentation, and imaging findings. Furthermore, we discuss unusual infections and conditions that should be considered in cases of persistent or atypical genital inflammation as well as the differential diagnosis required to distinguish them from infectious causes.

## Female genital organs: pelvic inflammatory disease

PID is characterized by inflammation of female reproductive organs, including the uterus, fallopian tubes, and ovaries. It is typically caused by an ascending infection originating in the vagina or cervix. Infection of the fallopian tubes and uterus often occurs simultaneously and has the potential to spread to the ovaries and peritoneum. Severe forms of PID include tubo-ovarian abscess (TOA), peritonitis, and systemic illness [1, 2]. Sexually transmitted infections (STIs), such as those caused by Chlamydia trachomatis and Neisseria gonorrhoeae are commonly implicated as causative agents in approximately one-third to half of PID cases [3]. Recent data also suggest a potential role of Mycoplasma genitalium, which may be associated with milder symptoms [4]. Other contributing factors include intrauterine surgery, intrauterine devices (IUDs), delivery, and endometriosis. According to the National Health and Nutrition Examination Survey 2013–2014 cycle, the estimated lifetime prevalence of self-reported PID is 4.4% in sexually experienced women of reproductive age in the United States [4]. Identification of specific bacteria causing PID is typically accomplished through polymerase chain reaction testing or culture of cervical secretions. The signs and symptoms of PID are non-specific and can vary from asymptomatic to severe. Common clinical presentations include lower abdominal pain, irregular vaginal bleeding, vaginal discharge, cervical motion tenderness, fever, and dyspareunia. Early diagnosis and treatment are crucial to reduce the risk of long-term complications, including infertility. In sexually active young women at risk of STIs, presumptive treatment should be initiated if they present with pelvic or lower abdominal pain and no identifiable cause other than PID [3].

Ultrasonography (US) is often used to diagnose PID. Computed tomography (CT) is useful in the initial diagnosis when investigating the cause of abdominal pain. Magnetic resonance imaging (MRI), on the other hand, is usually an additional examination for the specific diagnosis of PID. In a recent meta-analysis, contrast-enhanced CT had a sensitivity of 0.79 and specificity of 0.99, while MRI had a sensitivity of 0.95 and specificity of 0.89 [5]. It was also reported that when DWI was added to MRI, the sensitivity increased from 90.7 to 98.4% and the specificity remained the same at 93.3% [5].

**Fig. 1:**
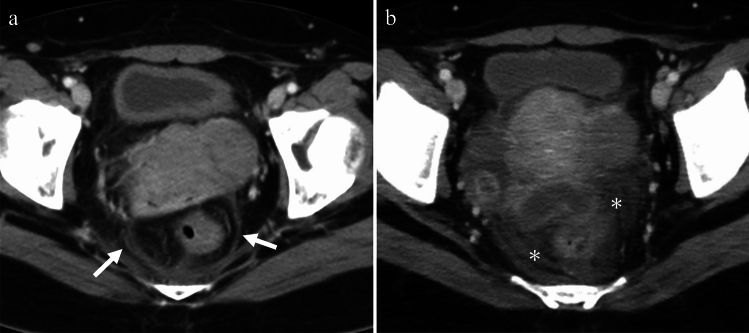
Woman in her 40 s with lower abdominal pain who tested positive for Chlamydia and Syphilis Thickening of the uterosacral ligament (arrows in **a**) and increased haziness of the presacral and perirectal fat planes (asterisks in **b**) are observed on contrast-enhanced CT. These are the general findings in pelvic inflammatory diseases

Imaging findings in PID are often non-specific, ranging from no findings to the formation of an adnexal mass, and the typical CT findings of PID include thickening and enhancement of the fallopian tubes, thickening of the uterosacral ligaments, an indistinct uterine and adnexal border, and pelvic fat stranding [6] (Fig. 1).

### Cervicitis

**Fig. 2:**
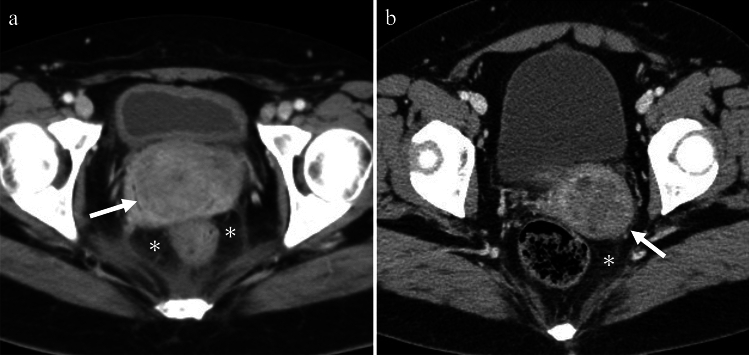
**a** Woman in her 40 s with lower abdominal pain who tested positive for Chlamydia Enlarged edematous uterine cervix (arrow) with fat stranding (asterisks) on contrast-enhanced CT indicates cervicitis. **b** Woman in her 40 s with abdominal pain who tested positive for Chlamydia Multiple cysts with heterogeneous attenuation (arrow), indicating cystic cervicitis are observed. A slight fat haziness (asterisk) is also observed

Cervicitis is characterized by cervical inflammation. Cervicitis is primarily diagnosed based on clinical findings, and no specific imaging findings are associated with this condition. However, imaging findings of cervicitis have been reported, including an enlarged and abnormally enhancing or hyperemic endocervical canal, with or without parametrial fat stranding visible on CT or MRI (Fig. 2a); free fluid may also be observed in the cul-de-sac [6]. Cystic cervicitis is a distinct form of cervical inflammation, and its symptoms may include jelly-like vaginal discharge. On imaging, cystic cervicitis typically presents as round, multicystic lesions located centrally within the cervix (Fig. 2b). Due to the presence of hemorrhage and infected material, the signal intensity on pre-contrast T1-weighted images may vary from low to high [7].

### Endometritis, uterine empyema (pyometra)

**Fig. 3:**
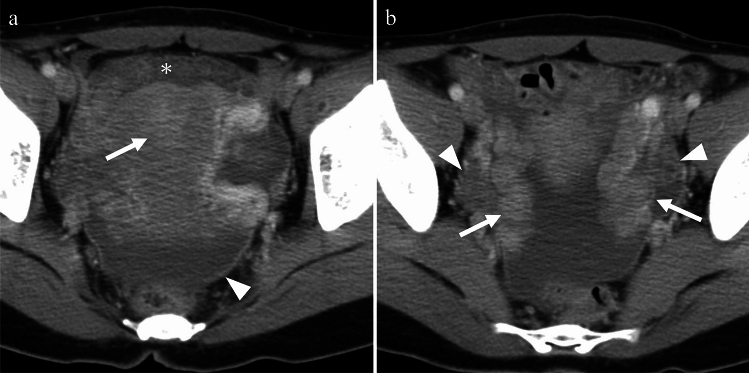
Woman in her 40 s with lower abdominal pain who tested positive for Chlamydia. **a** Contrast-enhanced CT revealing endometrial enhancement (arrow), indicating potential endometritis. Stranding of the anterior pelvic fat planes (asterisk) and the presence of complex free fluid, and peritoneal thickening (arrowhead) in the dependent areas are also noted. **b** Bilateral tubal thickening exceeding 5 mm (arrows) on contrast-enhanced CT is consistent with salpingitis. The ovaries exhibit a polycystic appearance (arrowheads) with mild haziness and hyperemia, suggestive of oophoritis

Endometritis refers to the inflammation or infection of the endometrium, the lining of the uterus. The diagnosis of endometritis is primarily based on clinical findings. In contrast, uterine empyema is a condition in which the uterine cavity is filled with purulent content [6]. It is often a consequence of endometritis and can be caused by various factors, such as benign conditions, including endometrial polyps, senile endometritis, idiopathic conditions leading to cervical stenosis, or postoperative infections. Uterine empyema is commonly observed in postmenopausal women and is typically asymptomatic; its incidence increases with age and decreased activity, with incontinence being a significant risk factor [6]. CT findings of endometritis are non-specific and may include a normal-appearing endometrium, an enlarged uterus, and fluid accumulation in the endometrial canal. Abnormal enhancement of the endometrium was also observed [6] (Figs. 3a and 5a). Uterine empyema is characterized by the distention of the uterine cavity with complex fluid, and imaging findings may include gas bubbles and air-fluid levels [6] (Fig. 5b).

### Salpingitis, tubal empyema (pyosalpinx)

**Fig. 4:**
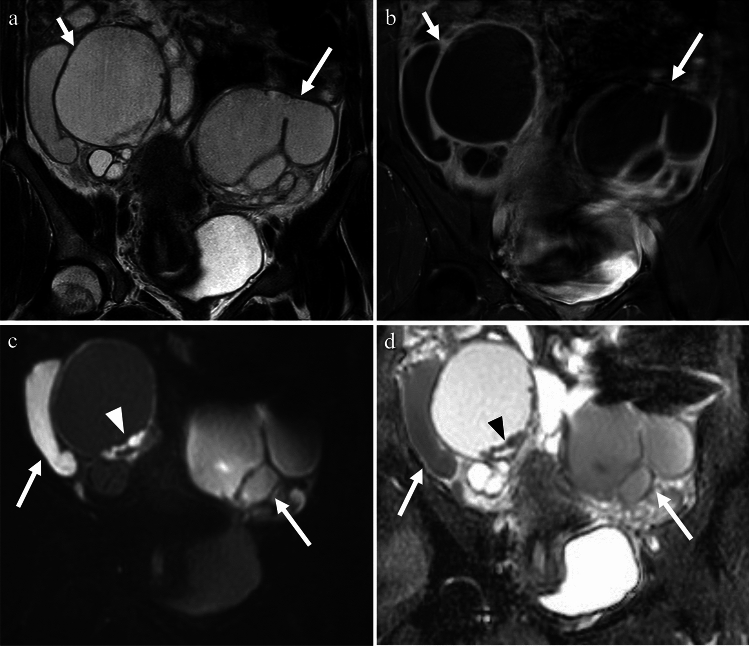
Woman in her 20 s with fever and lower quadrant pain who tested positive for Chlamydia. **a** T2-weighted image shows cystic lesions in bilateral adnexa (arrows), and tubular structures and plica indicating fallopian tubes. The contents of the fluid have a lower signal intensity than urine. **b** Thickening and enhancement of the cyst wall (arrows) seen on fat-saturated contrast-enhanced T1-weighted image are indicative of tubal empyema. **c**, **d** Some of the contents show a prominent high signal on DWI with decreased ADC (arrows), indicating pus. Debris with strong diffusion restriction is also observed (arrowheads)

Salpingitis is the most common early acute form of PID and refers to the inflammation of the fallopian tubes. It is primarily caused by STIs, and if left untreated, can progress to tubal empyema, a condition characterized by the accumulation of pus in the fallopian tubes. Salpingitis can be categorized as acute or chronic. Acute salpingitis is an infection of the fallopian tubes, in which pus may accumulate in one or both tubes. Chronic salpingitis refers to persistent inflammation of the fallopian tubes that results in adhesions within the tubal lumen. This can lead to tubal damage, pelvic pain, and infertility. Women with chronic salpingitis are at an increased risk of ectopic pregnancies (30–40%) and infertility (40–50%) [6]. The diagnosis of salpingitis relies primarily on clinical findings. Imaging findings of salpingitis are non-specific and may include a normal appearance to thickening of the fallopian tubes (usually ≥ 5 mm), pelvic fat infiltration or haziness, and pelvic edema [6, 8, 9] (Fig. 3b). Tubal empyema is characterized by fluid-filled fallopian tubes, with thickening and enhancement of the tubal wall (Fig. 4). Hydrosalpinx refers to fimbrial obstruction and distention of the fallopian tubes with non-purulent fluid. Although often asymptomatic, it can cause chronic pelvic pain, dyspareunia, and infertility [6, 10].

### Oophoritis

Oophoritis is a condition characterized by the extension of infection from the fallopian tubes to the ovaries. Inflammatory exudates spread to the ovary or periovarian region, leading to swelling of the ovarian stroma, surrounding edema, hyperemia, and a polycystic appearance on imaging. Oophoritis is often represented by polycystic ovaries with multiple small follicles measuring 2–10 mm and an ovarian short-axis diameter > 3 cm (Figs. 3b and 7a). However, there is an overlap with a normal ovarian appearance, making it challenging to diagnose oophoritis based solely on ovarian findings [8].

### Tubo-ovarian abscess

**Fig. 5:**
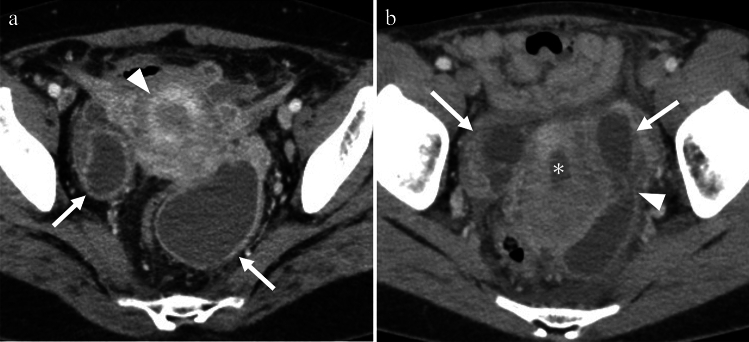
**a** Woman in her 40 s with fever and lower abdominal pain who tested positive for Chlamydia. Contrast-enhanced CT image demonstrates abnormal enhancement of the endometrium and hyperemia of the inner myometrium (arrowhead), indicating endometritis. Moreover, fluid collections with thick enhancing walls are visualized in both adnexas (arrows), suggesting tubo-ovarian abscesses. **b** Woman in her 40 s with fever and lower abdominal pain, positive for Gram-positive and Gram-negative rods The presence of complex fluid causing distention of the uterine cavity (asterisk) on contrast-enhanced CT is indicative of uterine empyema. In addition, the accumulation of fluid with thick enhancing walls in both adnexa (arrows) suggests the presence of tubo-ovarian abscesses. Furthermore, there is evidence that the left TOA has perforated the abdominal cavity (arrowhead). A fatty haziness of the parametrium is also observed.

**Fig. 6:**
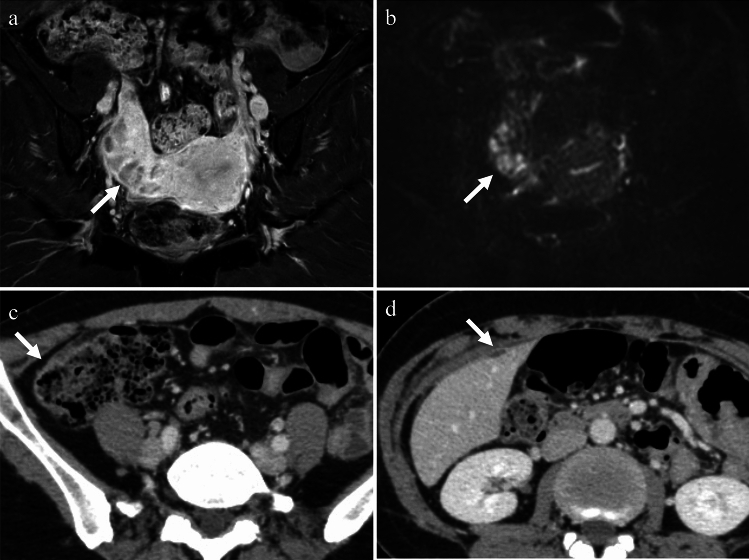
Woman in her 30 s with fever and right lower quadrant pain who tested positive for Chlamydia. **a** Fat-saturated contrast-enhanced T1-weighted image showing swollen and well-enhanced right adnexa (arrow), along with the presence of multiple cysts. **b** Cyst contents exhibit markedly restricted diffusion (arrow), suggesting the presence of pus and tubo-ovarian abscesses on diffusion-weighted image. **c** Thickening and enhancement of the peritoneum (arrow) in the right lower abdomen on contrast-enhanced CT are indicative of peritonitis. **d** Localized fluid accumulation with a peripheral enhancing effect at the margins of the hepatic surface (arrow) is observed on contrast-enhanced CT, suggesting the presence of an abscess

Tubo-ovarian abscess (TOA) is a severe and complex complication of PID, occurring in approximately 15–34% of patients with PID [11, 12]. TOA is characterized by the accumulation of pus in the fallopian tubes and ovaries. Risk factors for TOA include STIs, ovarian endometriosis, and other conditions that cause inflammation of the reproductive organs. Imaging findings of TOA typically reveal a multilocular septate cystic mass in the adnexa with a thick, uniformly enhancing wall. There is often a loss of fat planes between the mass and adjacent pelvic organs. In addition, thickening of the uterosacral ligaments and presence of fluid in the cul-de-sac may be observed [6] (Figs. 5, 6a, b).

### Peritonitis

Pyometra, pyosalpinx, and TOA can cause peritonitis when an abscess ruptures or pus leaks from an infected organ. Peritonitis is a serious condition that requires urgent medical attention and typically involves antibiotics, and if necessary, surgical intervention. Delayed diagnosis and treatment can result in severe complications including sepsis and even death [2, 13]. Contrast-enhanced CT is a valuable tool for diagnosing peritonitis and determining its underlying cause. Findings associated with peritonitis include enhancement of the peritoneum with thickening, enhanced fascial planes, inflammatory changes in the mesentery and bowel wall, and intraperitoneal abscesses [13] (Fig. 6c, d).

### Fitz-Hugh–Curtis syndrome

**Fig. 7:**
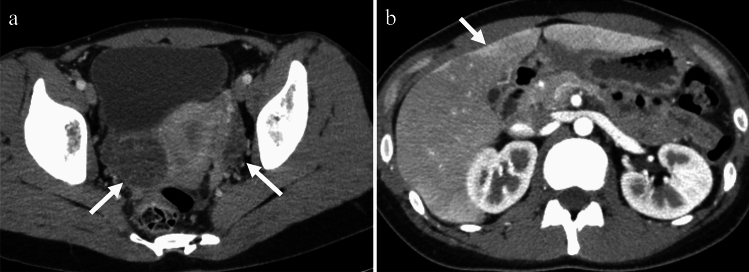
Woman in her 30 s with fever and right lower quadrant pain who tested positive for Chlamydia. **a** Contrast-enhanced CT reveals polycystic enlargement of the bilateral ovaries (arrows), indicating oophoritis. **b** Peripheral hepatic capsular hyperemia (arrow) is observed on arterial-phase contrast-enhanced CT, which is compatible with Fitz-Hugh-Curtis syndrome

Fitz-Hugh–Curtis syndrome is a rare complication of PID characterized by inflammation of the liver capsule and the formation of adhesions. The condition was named after the two physicians who first reported it in the 1930s [14]. The primary symptom of Fitz-Hugh–Curtis syndrome is sudden onset of abdominal pain in the right upper quadrant, which may be accompanied by right shoulder pain. The diagnosis of this syndrome can be challenging, as pelvic pain, vaginal discharge, and cervical tenderness are frequently absent or minimal, if present. This condition occurs when infectious bacteria travel from the pelvis to the liver capsule, bypassing pelvic structures. Contrast-enhanced CT findings associated with Fitz-Hugh-Curtis syndrome may include marked enhancement in arterial-phase, thickening of the liver capsule, fluid and fat stranding extending from the pelvis into the right upper quadrant through the paracolic gutter, loculated fluid around the liver, and gallbladder wall thickening [9, 15] (Fig. 7b). Fitz-Hugh–Curtis syndrome can also occur in men [14, 15].

### Other complications

Chronic inflammation can lead to the formation of scar tissue and adhesions within the affected organ as well as with surrounding organs. Adhesions within the bowel can cause bowel obstruction, whereas those within the ureter can result in hydronephrosis. Furthermore, chronic inflammation can give rise to thrombophlebitis, which involves the formation of blood clots within the ovarian veins. The combination of inflammation, impaired blood flow, increased blood coagulation, and vein compression contributes to the development of ovarian vein thrombophlebitis. If left untreated, this condition can pose a risk of pulmonary embolism, in which the blood clot travels to the lungs and blocks the pulmonary arteries [6].

### Unusual causes of pelvic inflammatory disease

#### Actinomycosis

**Fig. 8:**
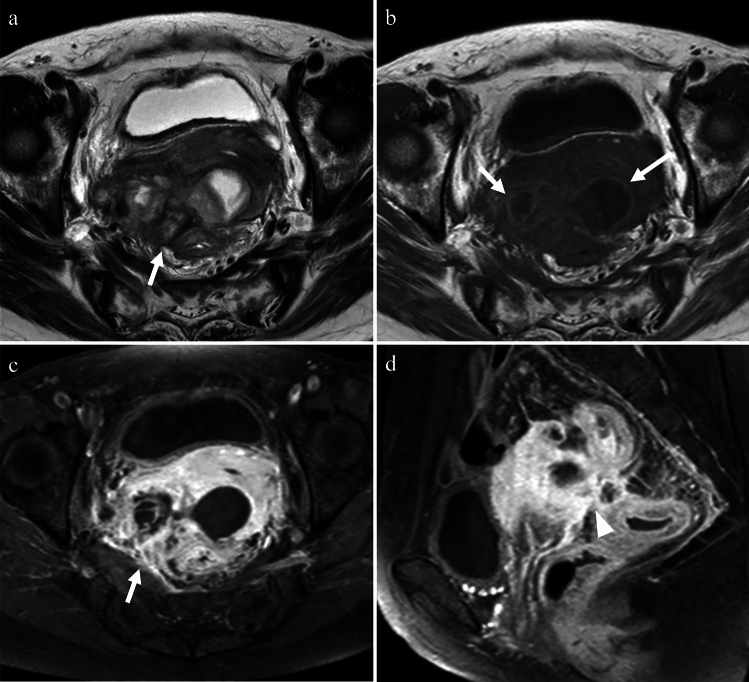
Woman in her 60 s with a history of intrauterine device use who presented with genital bleeding and tested positive for Actinomycosis. **a** A solid cystic mass, adhered extensively to the uterus, is observed with an indistinct border. The cystic contents exhibit heterogeneity on T2-weighted image. **b** The presence of rim-like high signal intensity at the cyst margins (arrows) on T1-weighted image indicates the presence of abscesses. **c** The solid component demonstrates strong enhancement (arrow) on fat-saturated contrast-enhanced T1-weighted image (FS-CE-T1WI). **d** Infiltration into the rectum (arrowhead) is evident on sagittal FS-CE-T1WI

Pelvic actinomycosis is a rare type of PID caused by a group of bacteria known as Actinomyces. These bacteria are non-motile, filamentous, gram-positive, anaerobic-to-microaerophilic organisms that form filamentous microcolonies. They do not produce spores, and exhibit slow growth [16]. Pelvic actinomycosis primarily affects the ovaries and fallopian tubes. Actinomycosis is often introduced through the presence of foreign bodies such as IUDs, and the infection may occur months or years after IUD removal [16]. Contrast-enhanced CT and MRI findings that suggest pelvic actinomycosis include a solid mass with strong enhancement and small abscesses within the mass that show rim enhancements. The presence of prominent fibrotic tissue in the affected area results in intermediate to low signal intensity on T2-weighted images for both the inflammatory strands and the solid component of the mass. Pelvic actinomycosis can also spread extensively across anatomic barriers such as the serosa and ligaments to nearby structures, such as the ureter, bladder, rectum, abdominal wall, and peritoneum. This can lead to the formation of a frozen pelvis, which resembles a pelvic malignancy or endometriosis [16, 17] (Fig. 8).

#### Tuberculosis

**Fig. 9:**
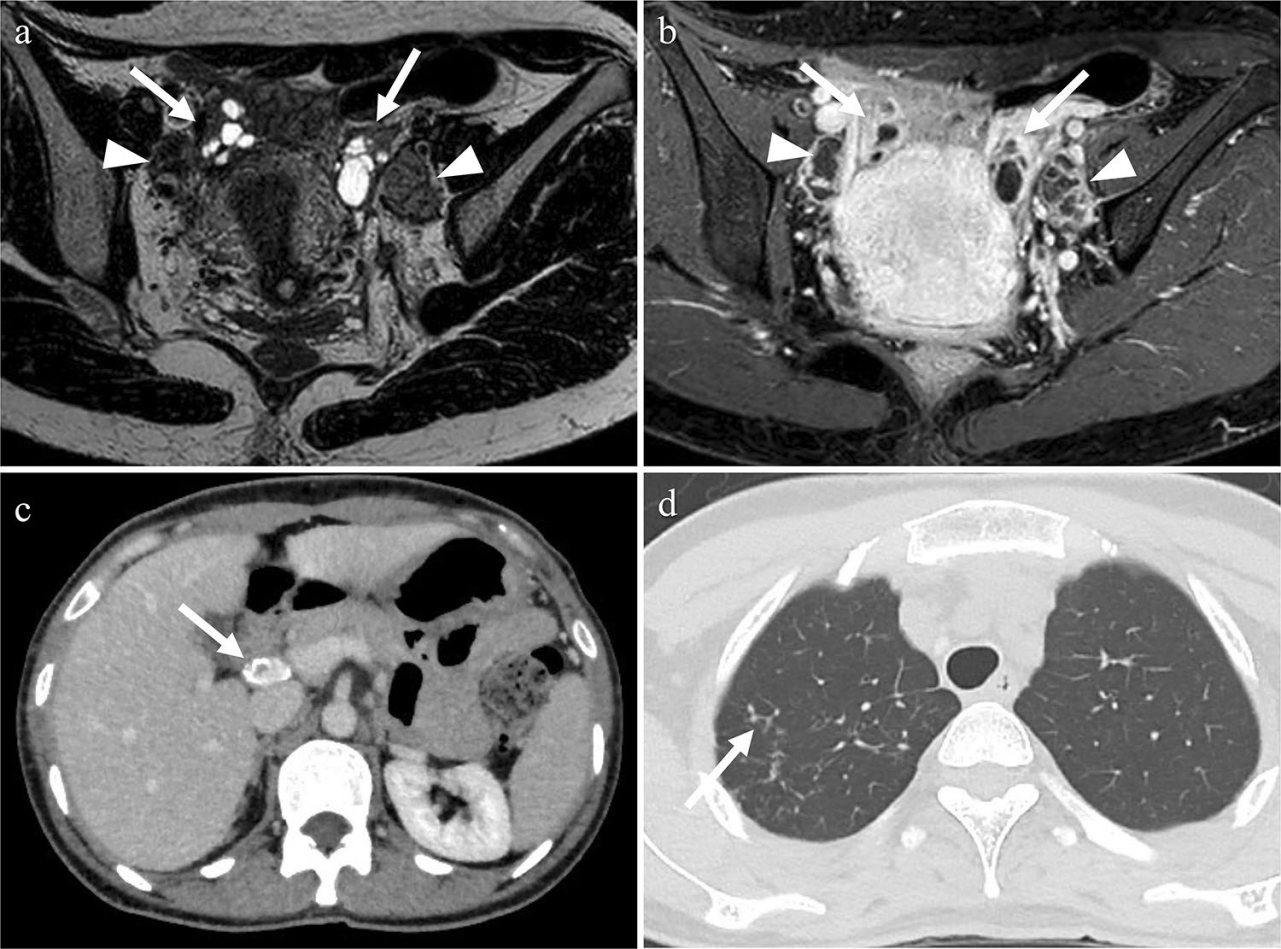
Woman in her 30 s with tuberculous tubo-ovarian abscesses. **a** Multiple cysts with septal thickening in both adnexa (arrows) and bilateral obturator lymph node swelling (arrowheads) are seen on the T2-weighted image. **b** The septa are strongly enhanced on fat-saturated contrast-enhanced T1-weighted image (arrows), indicating abscesses. Necrotic pelvic lymph nodes with a central contrast defect (arrowheads) are also demonstrated and tuberculosis is suspected. **c**, **d** Calcified hilar lymph node of the liver (arrow in **c**) and centrilobular nodules (tree-in-bud appearance) (arrow in **d**) in the right upper lobe of the lung on CT are also supportive of tuberculosis

**Fig. 10:**
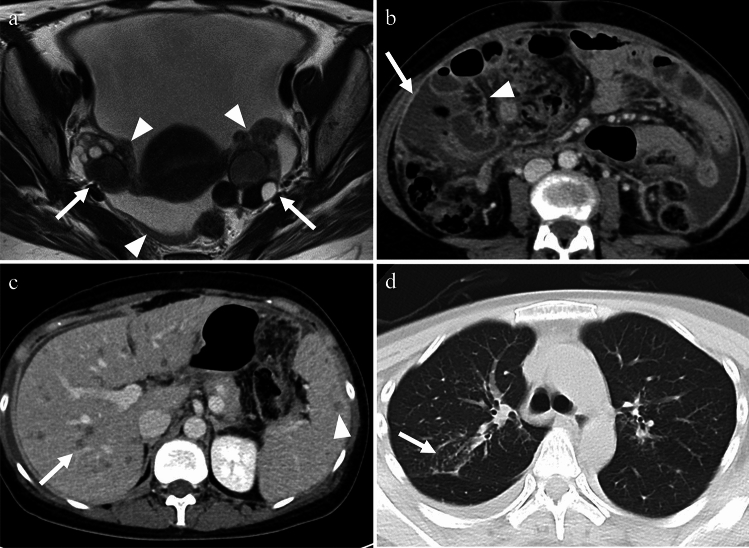
Woman in her 30 s with peritoneal tuberculosis. **a** On T2-weighted imaging, multilocular cystic tumors with heterogeneous signals suggestive of endometriotic cysts are observed in both ovaries (arrows). There is a significant amount of ascites and diffuse and smooth thickening of the peritoneum (arrowheads). Solid components were also present around the ovarian masses (arrowheads). **b** Contrast-enhanced CT showing diffuse thickening of the peritoneum (arrow) and mesentery (arrowhead). **c** Multiple small hypoattenuating nodules are scattered throughout the liver (arrow) and spleen (arrowhead) parenchyma on contrast-enhanced CT. **d** On chest CT, centrilobular granular nodules are identified in the right upper lobe (arrow), suggesting tuberculosis

Tuberculosis (TB) is caused by Mycobacterium tuberculosis. While TB primarily affects the lungs, it can also affect the female genital organs in approximately 1% of cases [18] and can cause infertility, menstrual irregularities, and pelvic pain. Genital TB, with vague symptoms and elevated serum carbohydrate antigen 125 levels, can mimic ovarian cancer in both imaging findings and clinical presentation. The treatment typically involves administration of a combination of antibiotics for several months to a year [18]. On imaging, cystic adnexal masses, or a combination of solid and cystic masses, typically bilateral, may be observed (Fig. 9). These masses are often accompanied by ascites, omental or mesenteric infiltration, and peritoneal thickening. These findings closely resemble those of peritoneal carcinomatosis associated with ovarian cancer, although, tuberculous peritonitis is usually more diffuse and smoother than cancerous peritonitis [19] (Fig. 10). Consequently, a definitive diagnosis is often made postoperatively. Calcifications may be present in adnexal masses on CT, suggesting TB, although they are not commonly observed, particularly during active inflammation. Lymph node enlargement is common, with necrotic lymph nodes suggesting TB lymphadenitis [17, 18]. Peripancreatic lymph node involvement along the branches of the celiac axis is a well-recognized manifestation of abdominal tuberculosis and reflects the distribution of lymphatic drainage from the small bowel and liver. Calcifications within enlarged lymph nodes may also seen [19].

## Male genital organs

Bacterial infections of the male genital organs typically occur retrogradely in the urethra. Bacteria can travel from the ejaculatory ducts through the vas deferens to reach the epididymis and eventually the testis. Infections can spread from the urethra to the prostate and seminal vesicles. These pathways may originate from STIs or urinary tract infections. Common symptoms associated with these infections include fever, dysuria (painful urination), pelvic pain, and urinary retention. Diagnosis is typically confirmed by urinalysis. Male genital infections cause severe complications, such as infertility and sexual dysfunction [20].

### Vasitis

**Fig. 11:**
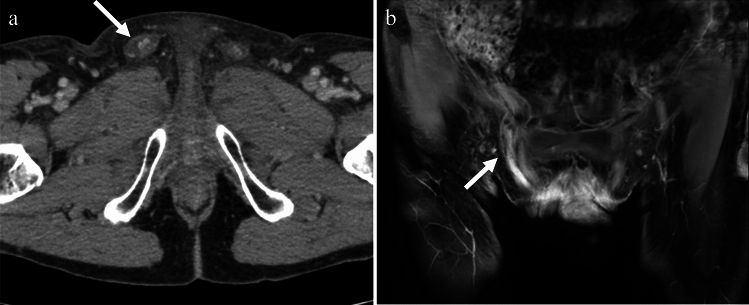
Man in his 60 s with right groin tenderness with unknown causative pathogen. **a** Contrast-enhanced CT reveals swelling in the right inguinal canal (arrow). **b** Coronal fat-saturated contrast-enhanced T1-weighted image reveals conspicuous hyperenhancement of the right spermatic cord (arrow), suggestive of vasitis

The vas deferens, also known as the ductus deferens, is a component of the spermatic cord. It originates at the caudal end of the epididymis and transports sperm from the epididymis to the ejaculatory duct. Vasitis, also referred to as deferentitis or funiculitis, is an acute inflammation that causes painful swelling of the groin. It is commonly observed in patients who have undergone procedures involving the manipulation of the vas deferens, such as vasectomy, prostatectomy, or herniorrhaphy. Infectious vasitis is often associated with cystitis, epididymis, or prostatitis [21]. US is considered the gold standard for excluding conditions such as epididymitis, orchitis, and other diseases. The normal appearance of the vas deferens on US is an anechoic or hypoechoic tubular structure with no detectable internal blood flow [22]. In contrast, vasitis shows a heterogeneously hypoechoic mass within the inguinal canal with increased vascularity on color Doppler US. However, US is less sensitive for distinguishing vasitis from incarcerated inguinal hernias. CT is more effective in differentiating between these two conditions and can reveal unilateral edema and abnormal enhancement of the spermatic cord [23] (Fig. 11). Some have reported that MRI is a preferred modality over CT because it detects abnormal signals in and around the vas that indicate edema and inflammation [24].

### Epididymitis, orchitis, epididymo-orchitis

**Fig. 12:**
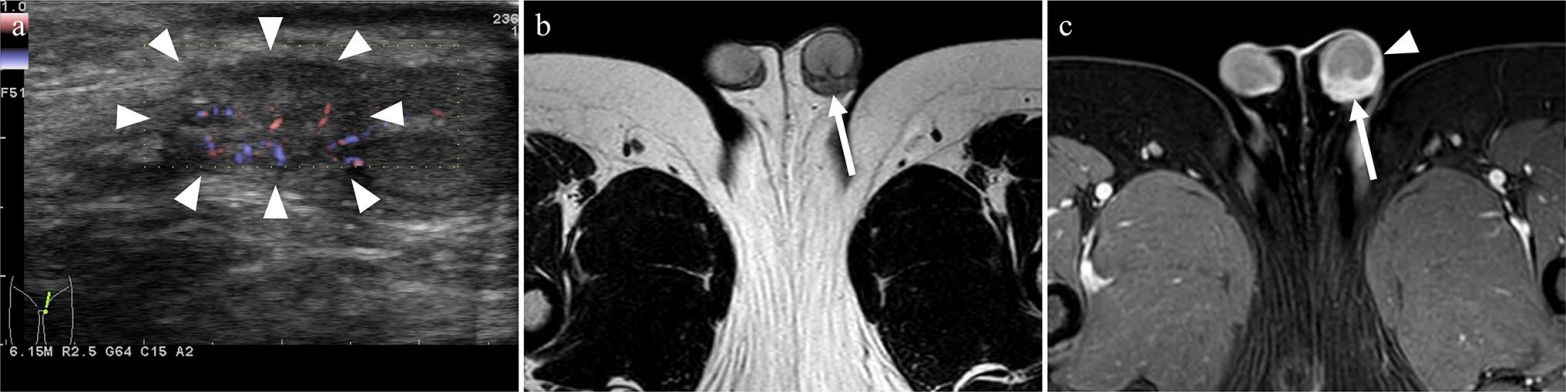
An 8-year-old boy with acute scrotal pain of unknown cause. **a** Color Doppler US showing an enlarged epididymis (arrowheads) with increased blood flow, corresponding to hyperemia. **b** On the T2-weighted image, the left epididymis is enlarged (arrow) compared to the opposite side. **c** On the fat-saturated contrast-enhanced T1-weighted image, the left epididymis appears more intensely enhanced (arrow) than the opposite epididymis, with noticeable enhancement of the scrotal wall (arrowhead). These findings were indicative of epididymitis

**Fig. 13:**
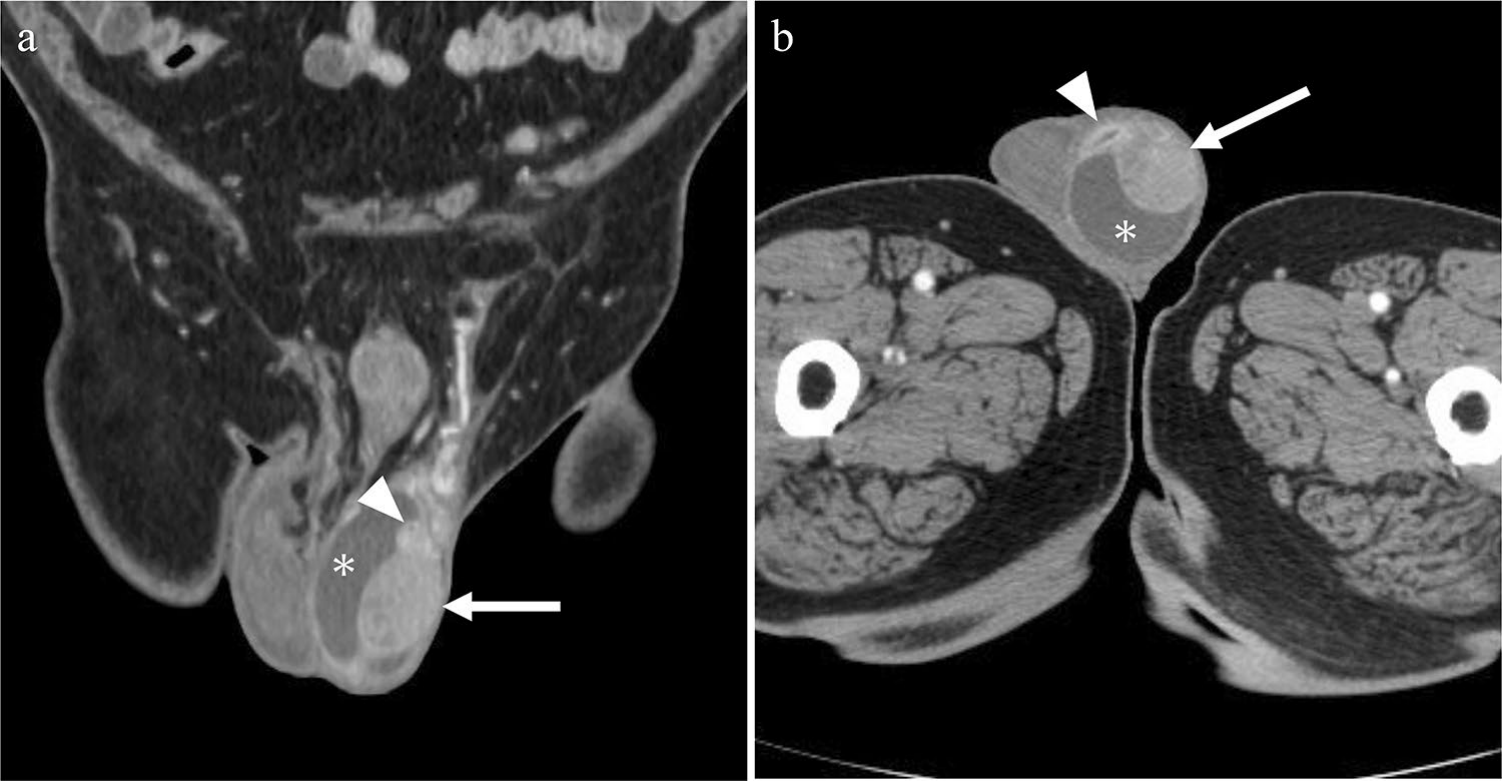
Man in his 70 s with fever who tested positive for Gram-positive bacilli. **a** Coronal contrast-enhanced CT shows enlargement and strong enhancement of the left epididymis (arrowhead) and testis (arrow), accompanied by thickening of the scrotal wall and reactive hydrocele (asterisk). These findings are indicative of epididymo-orchitis. **b** The axial plane reveals an abscess formation (arrowhead) is seen in contact with the testis (arrow) and hydrocele (asterisk)

Epididymitis, orchitis, and epididymo-orchitis are the most common causes of acute scrotal pain and involve inflammation of the epididymis, testes, or both. The infection typically spreads in a retrograde manner, starting from the tail of the epididymis, progressing to the head, and eventually to the testis. In some cases, orchitis can be caused by viral infections, with mumps being the most commonly associated viral cause; however, such cases are rare in developed countries owing to vaccination [25]. Recent studies have shed light on the mechanisms of mumps virus infection, interactions with host cells, and the resulting inflammatory response in testicular cells [26]. In sexually active men aged < 35 years, the most common pathogens are Chlamydia trachomatis and Neisseria gonorrhoeae. In other age ranges, organisms that typically contaminate the urinary tract, such as Escherichia coli and Pseudomonas species, are more common [20, 25]. US findings of epididymitis include an enlarged and hypoechoic epididymis due to edema. Reactive hydrocele and thickening of the scrotal wall may also be observed. Color Doppler US shows increased blood flow corresponding to hyperemia. When the infection spreads to the testis, the testicular parenchyma also exhibits heterogeneous echogenicity with increased blood flow [25]. On contrast-enhanced imaging, asymmetric enhancement of the spermatic cord vessels is indicative of ipsilateral infectious vasitis, epididymitis, or orchitis. In epididymitis, a strong enhancement of the epididymis due to hyperemia is usually observed [20, 27] (Fig. 12). Acute orchitis is indicated by asymmetry with enlargement and increased contrast enhancement of the affected testes; vasitis and epididymitis are often associated (Fig. 13). MRI has superior soft-tissue contrast resolution and can provide more valuable diagnostic information in differentiating other conditions such as testicular tumors. In addition, MRI is better at demonstrating testicular inflammation, as it shows decreased T1 signal intensity and increased T2 signal intensity compared to that in normal testes. The affected areas may exhibit intense and homogeneous enhancement or demonstrate the characteristic “tiger skin” post-contrast pattern, corresponding to preserved septa [20]. If left untreated, epididymitis, orchitis, and epididymo-orchitis can lead to complications, such as abscess formation (Fig. 13b), pyocele (collection of pus between the layers of the tunica vaginalis), and testicular ischemia. An abscess appears as a focal, complex fluid collection with no internal vascularity but with peripheral hyperemia. Pyocele is characterized by a complex collection of debris and septa. Testicular ischemia occurs when epididymal edema compresses the spermatic cord and restricts blood flow through the testicular vessels [20].

### Prostatitis

**Fig. 14:**
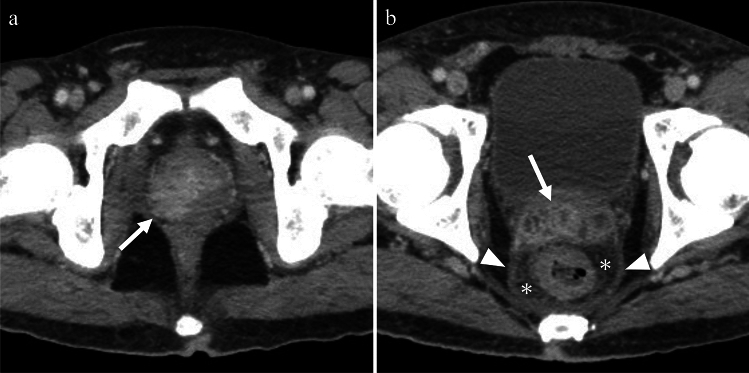
Man in his 30 s with leukemia presenting with fever with unknown causative pathogen. **a** The prostate is enlarged and shows marked enhancement predominantly on the right side (arrow) on contrast-enhanced CT, indicating prostatitis. **b** Contrast-enhanced CT showing diffuse wall and septal thickening of the seminal vesicle (arrow), thickening of the mesorectum (arrowhead), and increased haziness of the perirectal fat plane (asterisks). These indicate seminal vesiculitis

Prostatitis refers to infection or inflammation of the prostate gland. The most common route of infection is the ascending route, which is often associated with urinary tract infections, prostate biopsies, instrumentation, and in some cases, STIs. During digital rectal examination, the prostate gland is typically enlarged and tender. Approximately 10% of patients with acute prostatitis progress to chronic prostatitis, and an additional 10% develop chronic pelvic pain syndrome characterized by chronic pain in the perineum or lower abdominal regions [28]. The prostatic abscess is a rare but serious condition characterized by a collection of pus within the prostate gland. Imaging studies of prostatitis often show diffuse and asymmetric enlargement of the prostate, and contrast-enhanced images may reveal enhancement of the affected lesion and extraprostatic penetration [20] (Fig. 14a). Contrast-enhanced imaging findings of the prostatic abscess may demonstrate well-defined fluid collection with internal septations and peripheral enhancement [20].

### Seminal vesiculitis

Seminal vesiculitis refers to the infection or inflammation of seminal vesicles. Contrast-enhanced imaging often reveals diffuse wall and septal thickening with enhancement (Fig. 14b). During the acute to subacute phase, cystic dilatation of the seminal vesicles with surrounding hypervascularity or complications, such as abscess formation, may be observed. Seminal vesiculitis is commonly associated with spillage from prostatitis [20].

### Unusual causes of inflammation of the male genital organs

#### Tuberculosis

The most common site of male genital TB is the epididymis, which results from hematogenous spread or retrograde extension from the seminal vesicles and prostate. Typical findings of TB of the epididymis include an enlarged epididymis with calcification. In TB-endemic areas, an enlarged heterogeneous epididymis with predominant tail involvement can help differentiate TB from non-TB epididymitis [18]. Heterogeneity is likely due to the various stages of granuloma formation, including caseous necrosis and fibrosis. Color Doppler US may reveal a hypovascular central area, indicating caseous necrosis, with surrounding hyperemia, indicating peripheral granuloma formation. However, differentiating acute TB epididymitis from bacterial epididymitis is challenging [22].

Usually, the epididymis is initially involved, and if left untreated or undertreated, the infection can spread to the testes. Therefore, isolated testicular TB is rare and can simulate malignancy or infarction [18]. TB involvement of the testis varies and may involve the whole testis and present as orchitis, testicular abscesses, or hypoechoic nodules called tuberculomas. Differentiating TB from other forms of granulomatous orchitis or abscesses based on US features alone is difficult [30, 31]. On MRI, most lesions demonstrate heterogeneous intensity. Low signal intensity on T2-weighted images is typically associated with fibrosis, with more acute orchitis showing T2 hyperintensity. Contrast enhancement can range from no enhancement to homogeneous enhancement, with some cases showing ill-defined heterogeneous enhancement with an annular or multilocular pattern [18, 32]. Testicular and epididymal TB can present with or without an associated hydrocele, scrotal skin thickening, intra-scrotal extratesticular calcification, or scrotal abscess [32].

**Fig. 15:**
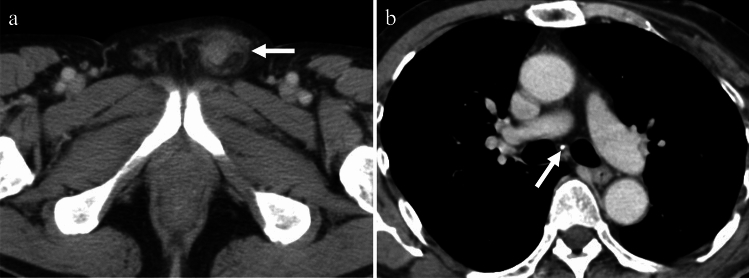
Man in his 60 s with tuberculous vasitis presenting with a left groin mass. **a** Contrast-enhanced CT showing swelling in the left inguinal canal (arrow) with increased peripheral fat density. **b** On chest CT, calcification corresponding to the mediastinal lymph nodes (arrow) is present, suggesting tuberculosis

TB infections of the vas deferens cause enlargement and heterogeneity of the vas deferens. Unilateral involvement is common, and the imaging features are usually similar to those of vasitis. The involvement can be focal, with little or no detectable flow on color Doppler US, and mimics a mass with central necrosis [18] (Fig. 15), whereas, non-TB acute vasculitis is usually more diffuse with increased vascularity.

Prostatic TB is usually asymptomatic and often detected incidentally in a transurethral resection specimen or prostate biopsy sample, as the imaging findings can mimic cancer. Prostatic TB can manifest as large abscesses in individuals infected with human immunodeficiency virus. US features include solitary or multiple hypoechoic nodules or masses resembling prostate cancer. Contrast-enhanced CT shows fluid-density collections with internal septa and enhancing rims. MRI findings are categorized into those with nodular and diffuse morphologies. The nodular type is characterized by the presence of caseous granulomas that exhibit low T2 signal intensity, restricted diffusion, and moderate enhancement. The diffuse type shows streaky lesions with low T2 signal intensity in the peripheral zone, creating a “watermelon appearance” [18].

Seminal vesicle involvement in TB usually occurs through contiguous spread from adjacent organs, typically from the prostate, and is rarely the primary site of origin. This results in the destruction of seminal vesicle, abscess formation, fibrosis, and calcification. Infertility due to azoospermia, hemospermia, or decreased ejaculatory volume may occur. CT and MR imaging reveal a diffuse wall and septal thickening with enhancement. Cystic dilatation of the seminal vesicles may be observed in the acute to subacute phases, followed by atrophy and hypointensity on T1- and T2-weighted images in the chronic phase. Abscess formation, cavitation, and caseous necrosis can also occur. Calcification may develop in burned-out TB lesions [18].

#### BCG-related infection

Infections resulting from intravesical Bacillus Calmette-Guérin (BCG) therapy for bladder cancer have been reported. BCG is a weakened live vaccine derived from Mycobacterium bovis and is commonly used in intravesical immunotherapy for intermediate- and high-grade bladder cancer. Despite its weakened state, BCG-contaminated urine can still cause local complications in the male genital system [25, 33]. The imaging findings of BCG-related infections in each organ resemble those of TB.

**Fig. 16:**
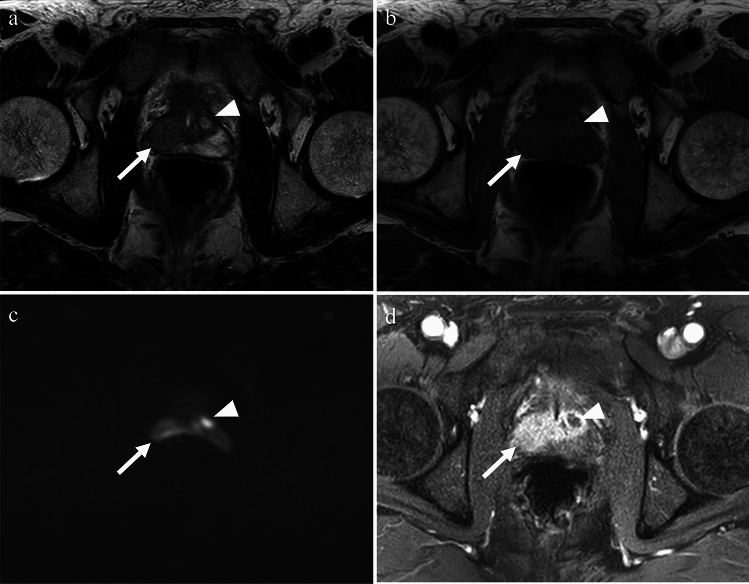
Man in his 60 s with BCG-related infection presenting with dysuria. **a** On T2-weighted image, a low signal area is observed on the right side of the prostate (arrow), and on the left side, a low signal nodule with high signal intensities in the central portion is noted (arrowhead). **b** On T1-weighted image, both show slightly higher signal intensities compared to the muscle (arrow and arrowhead). **c** On diffusion-weighted images, both exhibit strong high signal intensities (arrow and arrowhead). **d** After contrast administration, there is a diffuse enhancement on the right side, indicative of prostatitis(arrow), while the nodular lesion on the left side shows a contrast defect in the central portion, indicating an abscess (arrowhead)

BCG-associated granulomatous prostatitis is often asymptomatic. Histopathological evidence of granulomatous prostatitis has been found in asymptomatic patients, with an incidence of at least 88% after BCG therapy [33]. The imaging findings may mimic those of prostate cancer, with occasional abscess formation and prostate enlargement [33]. The involved areas appear hypoechoic on US, hypoattenuated on CT, and hypointense on T2-weighted images, and generally show increased signal intensity on T1WI and equal intensity with muscle, with enhancement and restricted diffusion due to high cellularity [34] (Fig. 16). It may be found incidentally on CT or 18F-fluorodeoxyglucose (18F-FDG) positron emission tomography (PET)-CT for bladder cancer follow-up [33].

BCG-related epididymo-orchitis is rare, occurring in less than 1% of cases as a complication of BCG treatment, and can manifest up to 10 years after the final intravesical BCG treatment [33]. Both the epididymis and testis are usually affected, although isolated epididymal involvement has also been reported [35]. The causal mechanism is thought to be the ejaculatory duct reflux [33]. US typically shows hypoechoic epididymal enlargement and development of heterogeneous hypoechoic masses corresponding to granuloma formation. Calcification may occur and bilateral involvement has been reported [22, 30]. Small hydroceles and scrotal thickening are usually observed. BCG infection can also progress to testicular abscess [33].

#### Zinner syndrome

**Fig. 17:**
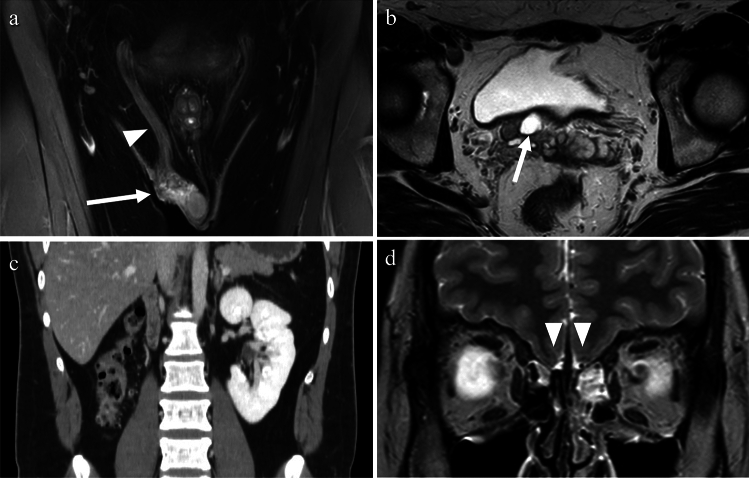
Man in his 20 s with Zinner and Kallmann syndrome presenting with recurrent right scrotal pain. **a** Fat-saturated contrast-enhanced T1-weighted image showing enlargement and enhancing effects in the right epididymis (arrow), with enhancement extending continuously along the spermatic cord (arrowhead), indicating epididymitis. **b** Cyst formation is also observed in the right seminal vesicle (arrow) on T2-weighted image. **c** Contrast-enhanced CT confirmed the right renal agenesis and a diagnosis of Zinner syndrome was made. **d** In addition, the absence of olfactory bulbs and olfactory grooves (arrowheads) on coronal T2-weighted image, combined with symptoms of hypogonadotropic hypogonadism and hyposmia, suggests Kallmann syndrome

Zinner syndrome is a rare congenital anomaly that can lead to recurrent epididymitis. It consists of a triad of Wolffian duct anomalies, including unilateral renal agenesis, ipsilateral seminal vesicle cysts, and ejaculatory duct obstruction. Inadequate drainage results in the enlargement of the seminal vesicle and the development of a cystic structure. The clinical symptoms are non-specific and may include frequent dysuria, epididymitis, perineal discomfort, and post-ejaculatory pain. The severity of symptoms is typically related to the size of the seminal vesicle [36] (Fig. 17). Some reports have suggested an association between Zinner syndrome and Kallmann syndrome [36]. Kallmann syndrome is a rare genetic disorder characterized by hypogonadotropic hypogonadism, accompanied by anosmia or hyposmia. MRI is the preferred imaging modality for evaluating the absence of the olfactory bulbs.

### Differential diagnosis of inflammation of the male genital organs

#### Vasculitis

**Fig. 18:**
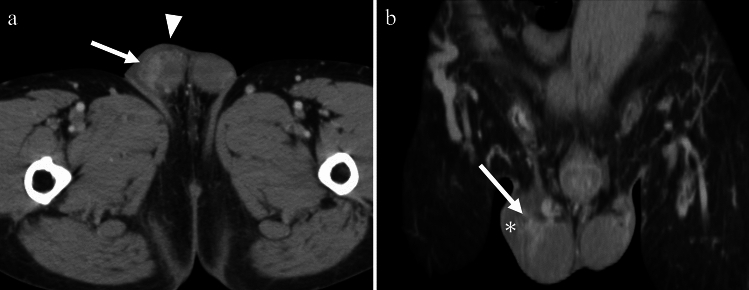
Man in his 30 s with Bechet’s disease presenting with right scrotal pain **a**, **b** On contrast-enhanced CT in axial and coronal sections, there is enlargement and strong enhancement of the right epididymis (arrows), accompanied by thickening of the scrotal wall (arrowhead in **a**) and reactive hydrocele (asterisk in **b**), indicating epididymitis

Behçet’s disease is a multisystem disorder of unknown origin, characterized by recurrent oral aphthous ulcers, ocular lesions, skin lesions, and genital ulcers. The disease predominantly affects individuals between the ages of 20 and 40 years, with a peak incidence at the age of 30 years and is particularly prevalent in areas along the Silk Road. Epididymitis is a symptom of Behçet's disease, with an incidence ranging from 0.6 to 32% [37]. Epididymitis is strongly associated with vulvar ulceration, skin lesions, arthritis, and central nervous system involvement, and is considered a relatively severe manifestation of the disease. In cases where the differential diagnosis of epididymitis has been excluded, and in the presence of recurrent episodes or the absence of bacteriuria or pyuria, epididymitis associated with Behçet’s disease should be considered [38] (Fig. 18).

Immunoglobulin A vasculitis, also called Henoch-Schönlein purpura, is the most common systemic vasculitis in children. The etiology is unknown and is characterized by leukocytoclastic vasculitis involving small vessels. Scrotal involvement is rare, and clinical manifestations include redness, swelling, and pain; US shows findings consistent with inflammation, including enlarged testes and epididymis and increased Doppler flow [39].

#### IgG4-related disease

**Fig. 19:**
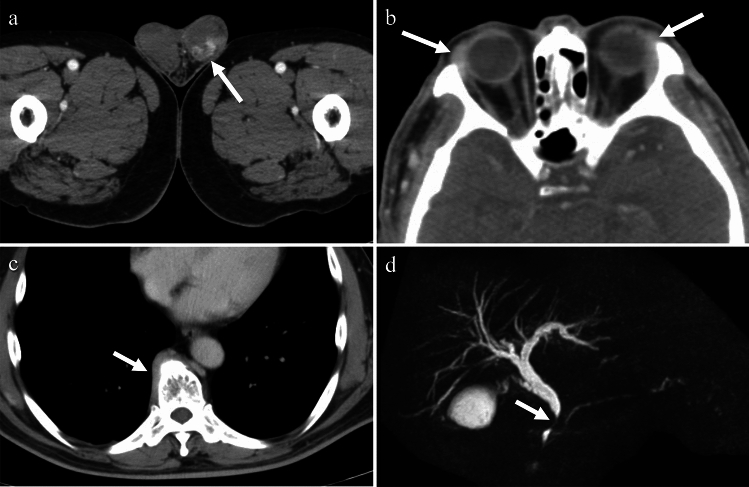
Man in his 60 s with IgG4-related disease presenting with left groin pain. **a** On contrast-enhanced CT, marked enhancement of the left epididymis (arrow) is accompanied by thickening of the scrotal wall, indicating epididymitis. **b** Enlargement of both lacrimal glands (arrows) is seen on contrast-enhanced CT. **c** Excessive soft tissue around the thoracic vertebrae (arrow) is shown on contrast-enhanced CT. **d** Lower bile duct stenosis (arrow) was observed on magnetic resonance cholangiopancreatography. These indicate abnormalities in various sites consistent with IgG4-related disease

Immunoglobulin G4 (IgG4)-related disease is a systemic inflammatory disease characterized by elevated serum IgG4 levels, copious infiltration of affected organs with IgG4-positive cells, and characteristic fibrosis called storiform fibrosis. IgG4-RD affects a broad range of organs such as the pancreas, lacrimal glands, salivary glands, kidneys, lungs, retroperitoneum, periaorta, skin, and lymph nodes. Prostatic involvement in IgG4-related disease has been also reported, and in most cases described in the literature, patients were retrospectively diagnosed with IgG4-related prostatitis based on histopathological findings following transurethral resection for symptoms of benign prostatic hyperplasia. A presumptive diagnosis is often made when symptomatic relief of benign prostatic hyperplasia is observed after glucocorticoid treatment [40, 41]. CT findings typically show diffuse enlargement of the prostate gland with homogeneous low attenuation, or the presence of nodular lesions within the prostate gland, with or without periprostatic soft-tissue infiltration [40, 41] (Fig. 19). PET imaging has also been used to support the diagnosis of IgG4-related prostatitis [41], showing an inverted V-shaped increase in FDG uptake in the prostate [42].

Testicular involvement is rare in this disease. The most common clinical presentation was an intra-scrotal mass, followed by swelling and pain. The right side was reported to be more involved than the left [43].

#### Sarcoidosis

**Fig. 20:**
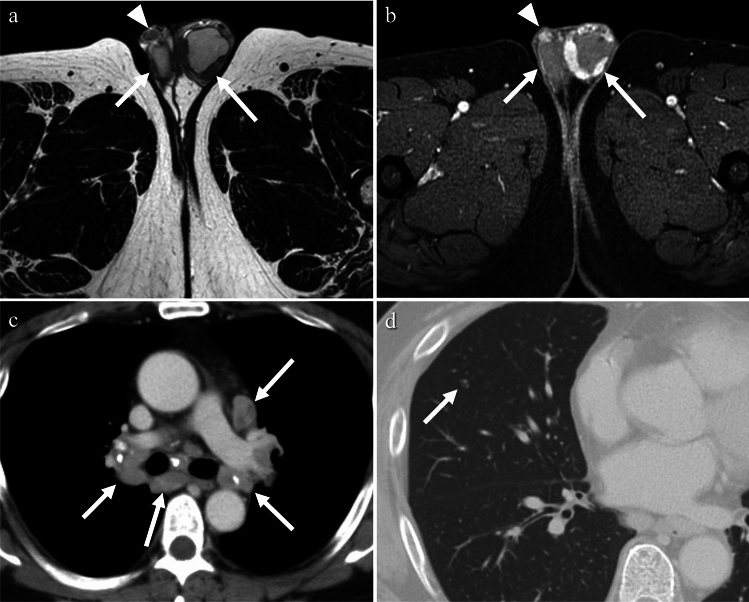
Man in his 40 s with sarcoidosis presenting with left inguinal tenderness. **a** Multiple nodules with low signal intensity are observed in both the testes (arrows) and epididymis (arrowhead) on T2-weighted images. **b** On fat-saturated contrast-enhanced T1-weighted images, the nodules exhibit strong enhancement (arrows and arrowhead). **c**, **d** A chest CT revealing calcified mediastinal and hilar lymphadenopathy (arrows in **c**) and centrilobular granular nodules (arrow in **d**). These findings led to a diagnosis of sarcoidosis

Sarcoidosis is a systemic disease characterized by the presence of non-caseating granulomas and the proliferation of epithelioid cells. It can affect multiple organs, and bilateral hilar lymphadenopathy is a common radiologic finding [44]. In intra-scrotal sarcoidosis, epididymitis is the most common manifestation, and testicular involvement is often associated with epididymitis, typically appearing as bilateral and multiple lesions. Patients with testicular sarcoidosis are typically asymptomatic and may present with a painless mass, or the mass may be incidentally discovered during the evaluation of pulmonary sarcoidosis [8, 44, 46]. The affected epididymis shows bilateral enlargement. Testicular lesions are observed as multiple nodules with decreased echogenicity on US, low signal intensity on T2-weighted images, and enhancement on contrast-enhanced images [35, 44–46] (Fig. 20). Increased uptake of 18F-FDG in the affected lesions has been reported [47].

## Conclusion

Inflammatory diseases primarily affect the reproductive organs and can lead to serious complications, such as abscesses, peritonitis, and infertility. Early diagnosis and appropriate treatment are important, and imaging modalities, such as US, MRI, and CT, play an important role in diagnosing the disease and its complications. They help to identify specific findings associated with various manifestations of inflammatory diseases. Awareness of these diverse etiologies, background diseases, and imaging findings is essential for accurate diagnosis of inflammation and related diseases.
